# Type I-E CRISPR-Cas Systems Discriminate Target from Non-Target DNA through Base Pairing-Independent PAM Recognition

**DOI:** 10.1371/journal.pgen.1003742

**Published:** 2013-09-05

**Authors:** Edze R. Westra, Ekaterina Semenova, Kirill A. Datsenko, Ryan N. Jackson, Blake Wiedenheft, Konstantin Severinov, Stan J. J. Brouns

**Affiliations:** 1 Laboratory of Microbiology, Department of Agrotechnology and Food Sciences, Wageningen University, Wageningen, The Netherlands; 2 Waksman Institute, Piscataway, New Jersey, United States of America; 3 Purdue University, West Lafayette, Indiana, United States of America; 4 Department of Immunology and Infectious Diseases, Montana State University, Bozeman, Montana, United States of America; 5 Department of Molecular Biology and Biochemistry, Rutgers, The State University, Piscataway, New Jersey, United States of America; 6 Institutes of Molecular Genetics and Gene Biology, Russian Academy of Sciences, Moscow, Russia; University of Geneva Medical School, Switzerland

## Abstract

Discriminating self and non-self is a universal requirement of immune systems. Adaptive immune systems in prokaryotes are centered around repetitive loci called CRISPRs (clustered regularly interspaced short palindromic repeat), into which invader DNA fragments are incorporated. CRISPR transcripts are processed into small RNAs that guide CRISPR-associated (Cas) proteins to invading nucleic acids by complementary base pairing. However, to avoid autoimmunity it is essential that these RNA-guides exclusively target invading DNA and not complementary DNA sequences (i.e., self-sequences) located in the host's own CRISPR locus. Previous work on the Type III-A CRISPR system from *Staphylococcus epidermidis* has demonstrated that a portion of the CRISPR RNA-guide sequence is involved in self versus non-self discrimination. This self-avoidance mechanism relies on sensing base pairing between the RNA-guide and sequences flanking the target DNA. To determine if the RNA-guide participates in self versus non-self discrimination in the Type I-E system from *Escherichia coli* we altered base pairing potential between the RNA-guide and the flanks of DNA targets. Here we demonstrate that Type I-E systems discriminate self from non-self through a base pairing-independent mechanism that strictly relies on the recognition of four unchangeable PAM sequences. In addition, this work reveals that the first base pair between the guide RNA and the PAM nucleotide immediately flanking the target sequence can be disrupted without affecting the interference phenotype. Remarkably, this indicates that base pairing at this position is not involved in foreign DNA recognition. Results in this paper reveal that the Type I-E mechanism of avoiding self sequences and preventing autoimmunity is fundamentally different from that employed by Type III-A systems. We propose the exclusive targeting of PAM-flanked sequences to be termed a target versus non-target discrimination mechanism.

## Author Summary

CRISPR loci and their associated genes form a diverse set of adaptive immune systems that are widespread among prokaryotes. In these systems, the CRISPR-associated genes (*cas*) encode for proteins that capture fragments of invading DNA and integrate these sequences between repeat sequences of the host's CRISPR locus. This information is used upon re-infection to degrade invader genomes. Storing invader sequences in host genomes necessitates a mechanism to differentiate between invader sequences on invader genomes and invader sequences on the host genome. CRISPR-Cas of *Staphylococcus epidermidis* (Type III-A system) is inhibited when invader sequences are flanked by repeat sequences, and this prevents targeting of the CRISPR locus on the host genome. Here we demonstrate that *Escherichia coli* CRISPR-Cas (Type I-E system) is not inhibited by repeat sequences. Instead, this system is specifically activated by the presence of *bona fide* Protospacer Adjacent Motifs (PAMs) in the target. PAMs are conserved sequences adjoining invader sequences on the invader genome, and these sequences are never adjacent to invader sequences within host CRISPR loci. PAM recognition is not affected by base pairing potential of the target with the crRNA. As such, the Type I-E system lacks the ability to specifically recognize self DNA.

## Introduction

There are several prokaryotic defense systems that confer innate immunity against invading mobile genetic elements, such as receptor masking, blocking DNA injection, restriction/modification (R-M) and abortive infection (reviewed in [1]–[3]). In addition, half of the bacteria, and most of the archaea, contain CRISPR-Cas (Clustered Regularly Interspaced Short Palindromic Repeats/CRISPR-associated) defense systems, unique in being the only adaptive line of prokaryotic defense (reviewed in [4]–[7]). CRISPR-Cas systems provide adaptive immunity to the host by incorporating invader DNA sequences into chromosomal CRISPR loci [8]–[11]. The 30–40 nt invader-derived DNA sequences are separated by host-derived similarly-sized repeat sequences. Adjacent to a CRISPR locus, a set of *cas* genes can often be found that encode the protein machinery essential for CRISPR-immunity. The *cas* genes occur in characteristic combinations that serve as a classification criterion of CRISPR-Cas systems into three major types [12]. In Type I and Type III systems the long precursor CRISPR RNA (pre-crRNA) is processed by CRISPR specific endoribonucleases into small CRISPR RNAs (crRNAs) that contain a repeat sequence flaked by portions of the adjacent CRISPR repeat sequence [13]–[18]. In some CRISPR-Cas subtypes the crRNA undergoes further processing at the 3′ end [19], [20]. In Type II CRISPR-Cas systems the pre-crRNA is processed by RNase III [21]. The processed crRNA molecules then remain bound to one or more Cas proteins to guide recognition and cleavage of complementary nucleic acid sequences [22]–[27].

With the exception of Type III-B CRISPR-Cas systems, which cleave RNA [23], [24],[28], all other characterized CRISPR-Cas systems appear to target DNA [27], [29]–[32] and hence require a mechanism to avoid aberrant cleavage of genomic DNA, i.e. a mechanism to discriminate the genomic “self” DNA of a CRISPR cassette from the invader “non-self” DNA. The absence of such discrimination leads to a suicidal autoimmune response [33]–[35]. In R-M systems this problem is solved by modification of the genomic DNA and cleavage of unmodified invader DNA only (reviewed in [3]). For CRISPR-Cas systems on the other hand, the mechanism(s) of self versus non-self discrimination is only partially understood.

For the Type III-A system of *Staphylococcus epidermidis* autoimmunity is prevented through a mechanism that relies on sensing base pairing between the 5′-handle (the repeat-derived sequence at the 5′-end of the crRNA) and the corresponding portion of CRISPR repeat [36]. The Type III-A CRISPR-Cas system consists of nine *cas* genes (*cas1*, *cas2*, *cas10*, *csm2*, *csm3*, *csm4*, *csm5*, *csm6*, *cas6*) and a CRISPR with type-8 repeats [37]. After a primary processing step of the pre-crRNA, the resulting crRNAs are further matured through ruler-based cleavage from the 3′ end, yielding 43 and 37 nt crRNA species [20]. These mature crRNA species guide one or more Cas proteins (possibly a Csm-complex) to target DNA [32], presumably through base pairing between the crRNA spacer sequence and the complementary protospacer sequence. However, CRISPR-interference is inhibited when, in addition to base pairing over the spacer sequence, the 5′-handle also base pairs with the protospacer-flanking sequence of the target DNA [36]. In this manner, self-targeting of the CRISPR locus is avoided by default, since self-targeting inevitably leads to full base pairing of the 5′-handle of the crRNA with the CRISPR repeat sequence from which it is transcribed. In particular, the presence or absence of base pairing at three positions downstream of the protospacer (positions −2, −3, and −4 relative to the 3′-end of the protospacer) is decisive in discriminating self from non-self [36]. The molecular details of how base pairing at positions downstream of the protospacer are sensed, and whether it involves Cas proteins, is currently unknown.

**Figure 1:**
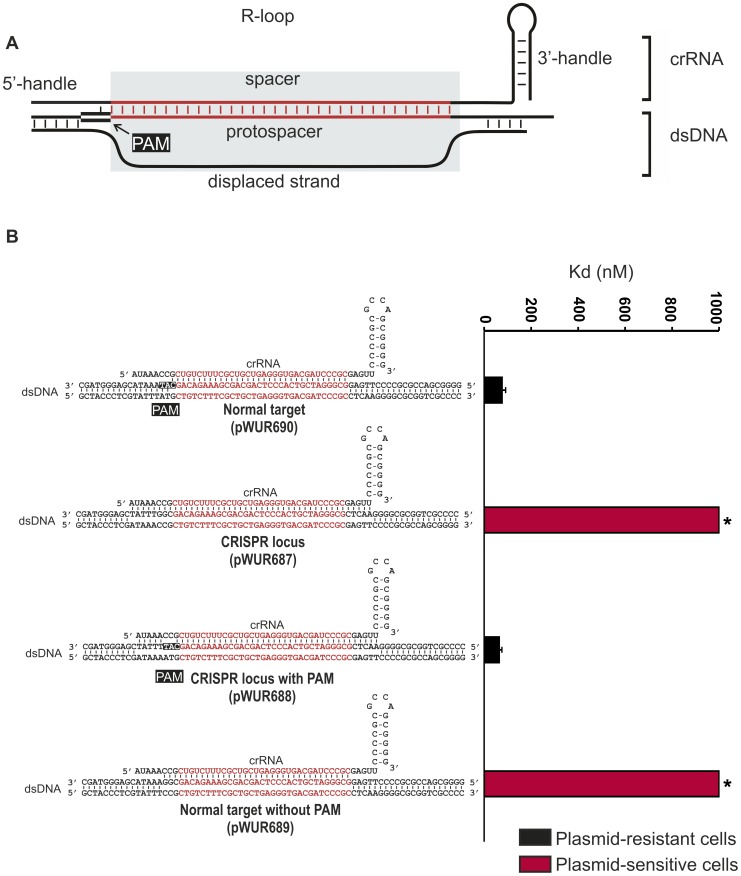
Potential base pairing between the crRNA repeat regions and protospacer flanking regions does not affect CRISPR-interference. **A**) Model of the R-loop formed by Cascade during dsDNA binding. **B**) Cells expressing WT g8-Cascade and Cas3 are resistant to plasmids containing the CAT PAM adjacent to the g8 protospacer (black bars, transformation efficiency 6.7±1.5×10^5^ cfu/µg DNA for plasmid pWUR690 and 6.8±0.9×10^5^ cfu/µg DNA for plasmid pWUR688), but are susceptible to plasmid transformation when the g8 protospacer is flanked by a CGG PAM, which is fully complementary to the 5′-handle (red bars, transformation efficiency 4.2±0.9×10^8^ cfu/µg DNA for plasmid pWUR687 and 4.5±0.8×10^8^ cfu/µg DNA for plasmid pWUR689). Transformation efficiency for a control pUC19 plasmid is 6.2±1.1×10^8^ cfu/µg DNA. The histogram shows the *in vitro* binding affinity of purified WT g8-Cascade for dsDNA containing the g8 protospacer flanked by sequences with a varying base pairing potential, as shown on the right. Asterisks indicate that the Kd value is >>1000 nM and the error bars represent the standard deviation of the mean.

**Figure 2:**
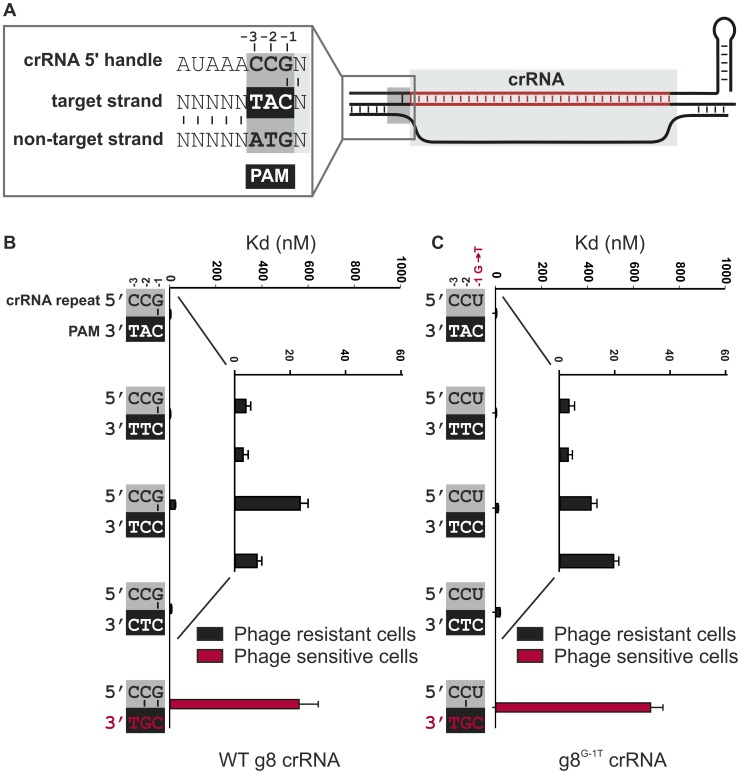
Base pairing at the −1 position is not required for CRISPR-interference. **A**) Model of the R-loop formed by Cascade during dsDNA binding. The nucleotide adjacent to the spacer sequence (the −1 position) has the potential to base pair with the first nucleotide of the PAM in the target strand of the DNA. **B**) Cells expressing WT g8-Cascade and Cas3 are resistant to M13 phage containing the CAT, CTT, CCT or CTC PAMs adjacent to the M13 protospacer (white font/black bars, e.o.p.<10^−4^), but not when containing the CGT PAM (red font/red bars, e.o.p. = 1). Note that in the figure the PAMs are oriented in 3′ to 5′direction to display base pairing potential with the last three nucleotides of the crRNA repeat. The *in vitro* binding affinity of purified WT g8-Cascade for dsDNA containing the g8 protospacer and each of the respective PAM mutants is shown in the adjacent histogram. **C**) Assays as in (B) using cells expressing the g8^G-1T^ CRISPR, Cascade and Cas3, show that cells are resistant to M13 phage containing the CAT, CTT, CCT or CTC PAMs adjacent to the g8 protospacer (white font/black bars), but not when containing the CGT PAM (red font/red bars). The *in vitro* binding affinity of purified WT g8^G-1T^-Cascade for dsDNA containing the g8 protospacer and each of the respective PAM mutants is shown in the adjacent histogram. In (B) and (C) error bars represent the standard deviation of the mean.

Intriguingly, Type I systems contain di- or tri-nucleotide conserved motifs (protospacer adjacent motifs (PAM)) downstream of protospacers opposite of the crRNA 5′-handle [38]–[40] (Figure 1A and 2A). In the Type I-E CRISPR-Cas system, PAM sequences are recognized by ribonucleoprotein complex Cascade during target DNA binding [29], [41]. The Type I-E system of *Escherichia coli* K12 consists of 8 *cas* genes (*cas3*, *cse1*, *cse2*, *cas7*, *cas5*, *cas6e*, *cas1*, *cas2*) and two CRISPR loci with type-2 repeats [37]. The ribonucleoprotein complex Cascade is composed of a 61 nt crRNA, and five different Cas proteins in an uneven stoichiometry: Cse1_1_Cse2_2_Cas7_6_Cas5_1_Cas6e_1_ [22]. Cascade efficiently binds target DNA through an R-loop formed between the 32 nt spacer sequence of the crRNA and the protospacer sequence [22] (Figure 1A), with a binding affinity that is strongly dependent on the presence of one of the four functional PAM sequences [29], [41]. Whereas R-loop formation by Cascade involves the entire protospacer sequence [22], it is unknown whether the PAM nucleotides can participate in base pairing with the crRNA and, if so, how this influences CRISPR interference. Due to the fact that the last nucleotide from the repeat is derived from the PAM sequence during spacer acquisition [8], [11], [42], this nucleotide in the crRNA invariably has the potential to base pair with the −1 position of the PAM, and therefore might be involved in R-loop formation [8]. In contrast, the −2 and −3 positions of the PAM lack base pairing potential with the 5′-handle of the crRNA (Figure 2A). The 5′-handles of other Type I systems and 3′-handles of Type II also display limited base pairing potential with their cognate PAMs (Table S1), in principle allowing for a differential base pairing mechanism that defines self versus non-self. For Type I-F CRISPR-Cas systems, potential base pairing between PAM sequences and the 5′-handle of the crRNA was recently shown to affect CRISPR interference [43], suggesting that self versus non-self discrimination in this subtype may depend both on sensing PAM identity and on sensing differential base pairing with the crRNA repeat.

In Type I-E systems it has been shown that a loop structure (L1) of the Cse1 subunit of Cascade specifically interacts with the PAM sequence, a process that is thought to destabilize the double-stranded DNA of the target to allow for strand invasion during R-loop formation [44]. Since self DNA of the CRISPR locus does not contain PAM sequences, this mechanism would specifically direct Cascade to target DNA only. However, the observation that target DNA containing a PAM mutant triggers Cascade-dependent primed spacer acquisition *in vivo* suggests that PAM authentication may not be absolutely required for R-loop formation [11]. Indeed, negatively supercoiled DNA containing a protospacer with a mutant PAM can still be bound by Cascade, albeit with a lower affinity than the same target with wild-type PAM [29]. In line with this, it was suggested that during phage infection Cascade can overcome the absence of a *bona fide* PAM when Cascade expression levels are high and that the target flanking sequences could participate in this discrimination event [44]. This suggests that a differential base pairing mechanism may play a role in self versus non-self discrimination by Type I-E CRISPR-Cas systems. In agreement with this, it was suggested that complementarity between the crRNA repeat and the protospacer flanking sequence inhibits CRISPR-interference in the Type I-E system of *Streptococcus thermophilus* [45]. The mechanistic basis of such a differential base pairing mechanism could lie in a perturbation of Cse1-mediated PAM recognition by base pairing interactions between crRNA repeats and the PAM.

To study whether a differential base pairing mechanism plays a role in self versus non-self discrimination by the Type I-E system of *E. coli* K12, we have systematically mutated both the crRNA repeats and the protospacer-flanking sequences and determined the effects of these mutations and their combinations on CRISPR interference *in vivo* and target binding *in vitro*. The results of our analysis demonstrate that discrimination of self from non-self by Type I-E CRISPR-Cas systems occurs through a mechanism that is independent of base pairing between these sequences. Hence, the principal mechanism by which Type I-E systems discriminate self from non-self appears to be solely Cse1-mediated and as such is fundamentally different from the differential base pairing mechanism employed by Type III-A systems. While the mechanism employed by Type III-A is best described as being based on self-recognition (self versus non-self), the mechanism of Type I-E systems is instead based on target-recognition (target versus non-target). While Type III systems can differentiate between targets and non-targets in the absence of a PAM, Type I-E systems are fully PAM-dependent and discrimination cannot take place in the absence of a PAM.

## Results

Self versus non-self discrimination by the Type III-A CRISPR-Cas system of *S. epidermidis* has been shown to rely on a differential base pairing mechanism [36]. As a result CRISPR-interference is specifically inhibited when protospacer sequences are flanked by CRISPR repeat sequences. To test whether this mechanism also applies to the Type I-E CRISPR-Cas system of *E. coli* K12, CRISPR-interference was tested against targets containing protospacers flanked by CRISPR repeat sequences. For these analyses, we have cloned the previously described g8 protospacer, from phage M13 [41], into the pUC19 plasmid and systematically mutated sequences adjacent to the protospacer. *E. coli* cells expressing Cascade, a g8 crRNA and Cas3 are resistant against transformation by a plasmid in which the g8 protospacer is flanked by a CAT PAM (Fig. 1B, pWUR690, approximately 1000-fold lower efficiency of transformation than a control pUC19 plasmid). In contrast, these cells are susceptible to plasmid transformation by plasmid pWUR687 in which the g8 protospacer is flanked by CRISPR repeat sequences (Figure 1B). However, the plasmid resistant phenotype can be restored by introducing a CAT PAM in the CRISPR repeat sequence flanking the protospacer (pWUR688), which alters the base pairing potential only at the −2 and −3 positions (Figure 1B). Plasmid pWUR689, which has the potential to base pair with g8 crRNA at positions −1, −2 and −3 (protospacer adjacent sequence is CGG) escapes CRISPR-interference from wild-type g8 crRNA expressing *E. coli* (Figure 1B). The observation that protospacer adjacent sequences complementary to the crRNA at positions −1, −2, and −3 avoid Cascade targeting suggest that base pairing at these positions may play a role in self avoidance.

To investigate whether avoidance of targeting is due to decreased binding affinities of Cascade for protospacers with mutations at the −1, −2, and −3 positions, we performed Electrophoretic Mobility Shift Assays using purified g8 crRNA-loaded Cascade. While high affinity binding could be demonstrated to dsDNA containing the g8 protospacer flanked by the CAT PAM (Figure 1B and S1), protospacers flanked by either CRISPR repeat sequences or a repeat-derived CGG sequence were bound with low affinity (Figure 1B and S1). This indicates that target versus non-target discrimination occurs at the level of Cascade affinity for dsDNA target sequences. Furthermore, the data also indicate that “self” DNA recognition may occur, as observed in Type III-A systems, through sensing differential base pairing between protospacer adjacent sequences and the 5′ handle of the crRNA.

To investigate if base pairing between the three nucleotides from the 5′-handle of the crRNA and the PAM is involved in discriminating self from non-self DNA we systematically mutated the corresponding nucleotides in the 5′-handle (i.e., −1, −2, and −3), and analyzed how these mutations affect CRISPR-based immunity against DNA targets flanked by various PAM sequences. Previously [29], four PAM sequences (CAT, CTT, CCT and CTC), have been reported to confer immunity on wild-type g8 crRNA expressing *E. coli* against phage M13 infection *in vivo*, and to give rise to high affinity DNA binding by g8 crRNA-bound Cascade *in vitro* (Figure 2B and Figure S2A). The last nucleotide of the 5′-handle of the crRNA (the −1 position) invariably has the potential to base pair with the PAM [8], while the −2 and −3 positions lack such base pairing potential (Figure 2A). The resulting configuration is distinct from the fully base-paired configuration that would form if base pairing in this region were the basis of self versus non-self discrimination.

To analyze whether base pairing at position −1 is required for CRISPR interference, a mutant CRISPR was constructed, yielding a g8 crRNA that lacks base pairing potential with the PAM at this position. This CRISPR, denoted g8^G-1T^ carries a G-to-T substitution at position −1, within the repeat sequence. SDS-PAGE analysis of purified Cascade complexes containing either mutant or WT crRNA shows that these complexes have the same apparent stoichiometry, thereby confirming the integrity of the complex (Figure S4A). In addition, isolation of crRNA from these protein complexes shows that crRNA biogenesis is unaffected by the introduced mutation (Figure S4B). Interestingly, despite the absence of base pairing at the −1 position, cells expressing the mutant crRNA maintain the ability to block infection by M13 phages containing each of the four functional PAM sequences (Figure 2C). Consistently, high affinity binding by g8^G-1T^ crRNA-containing Cascade to targets containing the g8 protospacer and the functional PAM variants was observed (Figure 2C and Figure S2B). However, as previously observed for the WT g8-crRNA-Cascade complex [29], a mutation at the −2 position of the PAM (i.e., C**G**T) neither confers resistance *in vivo* (efficiency of plaquing (e.o.p.) = 1) nor gives rise to high affinity DNA binding *in vitro* (Figure 2C, and Figure S2B). This PAM mutant potentially yields an additional base pair with the −2 position of the 5′-handle, both in the WT g8-crRNA-Cascade and the g8^G-1T^ mutant complex (Figure 2BC). Hence, it appears that a base pair at position −2 may be the signal that a protospacer is located in “self” DNA and therefore should not be targeted.

**Figure 3:**
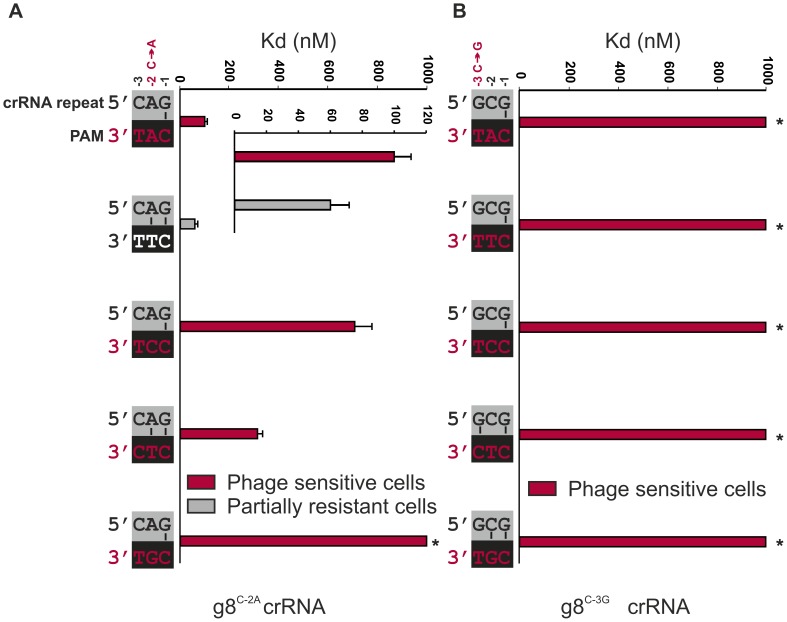
Base pairing at the −2 and −3 positions does not interfere with CRISPR-immunity. **A**) Cells expressing g8^C-2A^-Cascade and Cas3 are partially resistant to M13 phage containing the CTT PAM adjacent to the g8 protospacer (white font/grey bar, e.o.p.∼10^−2^), but not when containing the CAT, CCT, CTC or CGT PAM (red font/red bars). Note that in the figure the PAMs are oriented in 3′ to 5′direction to display base pairing potential with the last three nucleotides of the crRNA repeat. The *in vitro* binding affinity of purified WT g8^C-2A^-Cascade for dsDNA containing the g8 protospacer and each of the respective PAM mutants is shown in the adjacent histogram. **B**) Assays as in (A) using cells expressing the g8^C-3G^ CRISPR, Cascade and Cas3, show that cells are not resistant to M13 phage containing the CAT, CTT, CCT, CTC or CGT PAMs adjacent to the g8 protospacer (red font/red bars). The *in vitro* binding affinity of purified WT g8^C-3G^-Cascade for dsDNA containing the M13 protospacer and each of the respective PAM mutants is shown in the adjacent histogram. Asterisks indicate that the Kd value is >>1000 nM and the error bars represent the standard deviation of the mean.

To specifically test the role of base pairing at position −2 in CRISPR-immunity, we designed a synthetic CRISPR locus containing a C to A substitution at the −2 position of a CRISPR locus containing spacer sequences that target the g8 protospacer from M13 phage. The g8^C-2A^ CRISPR mutation results in a slight effect on Cascade assembly, as the bands corresponding to Cse1 and Cse2 have modestly lower and higher intensities on an SDS-PAGE, respectively, as compared to wild-type g8-crRNA-Cascade (Figure S4). However, g8^C-2A^ CRISPR RNA processing is unaffected (Figure S4). Importantly, the g8^C-2A^ crRNA-guided Cascade complex has a slightly reduced affinity (60±12 nM) for dsDNA targets that have a canonical CTT PAM sequence, which has the potential to base pair at the −2 position of the mutant crRNA (Figure 3A, white PAM). Despite the potential of the mutant Cascade complex to establish an additional base pair, a partially resistant phenotype (e.o.p.∼10^−2^) is observed against phages carrying the canonical PAM (Figure 3A), which is consistent with the *in vitro* DNA binding experiments (Figure 3A and Figure S3A). Targets containing non-canonical PAM sequences are bound with more reduced affinities by the g8^C-2A^ crRNA-guide Cascade complex and are not subject to CRISPR-interference *in vivo* (Figure 3A). The partial resistant phenotype of the g8^C-2A^ mutant that is observed in combination with the canonical PAM indicates that potential base pairing at both positions −1 and −2 does not serve as a trigger for a non-targeting response.

To probe the importance of base pairing at the −3 position, an additional CRISPR mutant was designed, denoted g8^C-3G^, which carries a C to G mutation at the −3 position of the CRISPR repeat. Again, complex formation and crRNA biogenesis were unaffected by the mutation (Figure S4). Although the potential for base pairing with most PAM sequences remains the same, a dramatic decrease in both resistance against M13 phage *in vivo* and DNA binding by g8^C-3G^-Cascade *in vitro* is observed (Figure 3B and Figure S3B).

**Figure 4:**
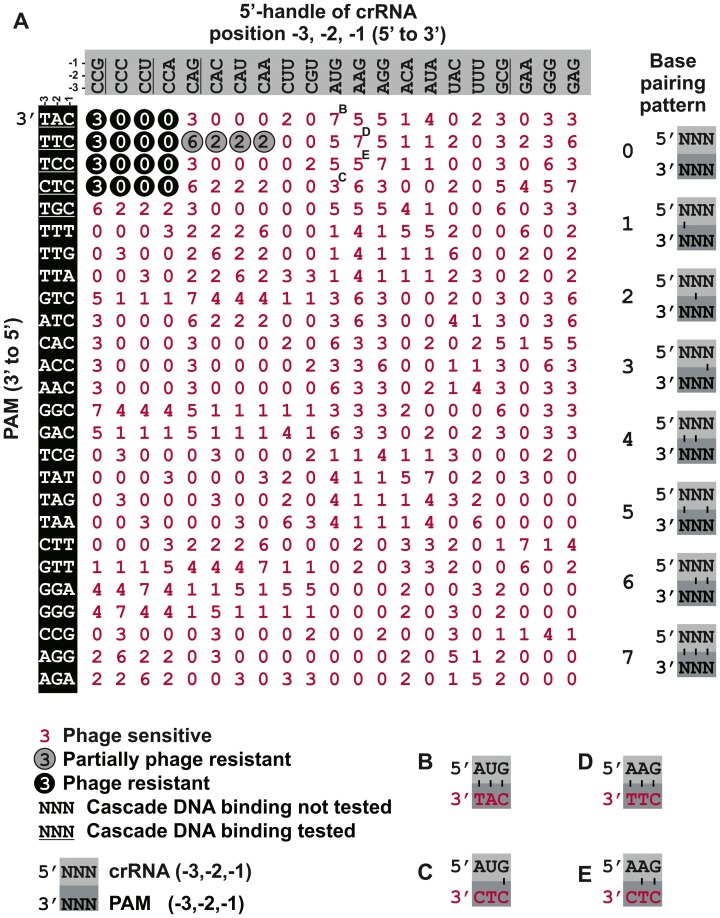
Synonymous mutations of the crRNA and the PAM do not affect self versus non-self discrimination. **A**) Infectivity of a library of M13 phage containing PAM mutants adjacent to the g8 protospacer was tested against cells expressing Cascade, Cas3 and g8 CRISPR containing mutations at the −1, −2 and −3 nucleotides of the CRISPR repeats. PAM mutations are shown on the left, with the PAM sequences indicated in the 3′ to 5′ direction. CRISPR repeat mutations at positions −1, −2 and −3 are indicated on the top in the 5′ to 3′ direction. Underscored sequences have been tested for binding affinity by EMSA. Base pairing potential between the PAM positions and the repeat is indicated using numbers (0–7) that correspond to a base pairing pattern that is shown in the panel on the right. A 0 signifies no base pairing, a 1 signifies base pairing at the −3 position, a 2 signifies base pairing at the −2 position, etc. Black circles with white digits indicate resistance against phage infection (e.o.p.<10^−4^), grey circles indicate partial resistance (e.o.p.∼10^−2^) and red digits without circle indicate susceptibility to phage infection (e.o.p. = 1), as determined by phage spot assays. Letters B, C, D, E indicate combinations shown in detail in the corresponding panels. **B**) Combination of g8^C-3AC-2T^ CRISPR and M13 phage with CAT PAM, gives rise to full base pairing and a lack of resistance (red font). **C**) Combination of g8^C-3AC-2T^ CRISPR and M13 phage with CTC PAM, gives rise to only base pairing at the −1 position and yields a lack of resistance (red font). **D**) Combination of g8^C-3AC-2A^ CRISPR and M13 phage with CTT PAM gives rise to full base pairing and a lack of resistance (red font). **E**) Combination of g8^C-3AC-2A^ CRISPR and M13 phage with CTC PAM gives rise to only two potential base pairs at the −1 and −2 positions and yields a lack of resistance (red font). Note in (A–E) PAMs are oriented in 3′ to 5′direction to display base pairing potential with the last three nucleotides of the crRNA repeat.

The combined results obtained with the three CRISPR mutants indicate that the repeat sequence itself rather than its base pairing potential with the protospacer flanking sequence affects PAM recognition. In order to have a more complete and unbiased analysis of the effects of adding or removing base pairing potential at positions −1, −2 and −3, we constructed 26 different PAM sequences adjacent to the g8 protospacer in the M13 phage genome (Figure 4A, white text on black background). All phages were viable as judged by their ability to infect host bacteria lacking the M13-targeting CRISPR (data not shown). The phages were tested for their ability to infect cells expressing each of the 21 different g8 crRNAs with mutated repeat sequences at positions −1, −2 and −3. Northern blot analysis showed that processing of mutant g8 crRNAs was unaffected (data not shown). The results reveal that only a small subset of CRISPR repeat mutants confer full phage resistance, and only in conjunction with the four previously validated functional PAM sequences (Fig. 4). When resistance was observed, it was independent of crRNA-PAM base pairing patterns, but rather appeared to be constrained by a limited number of allowed nucleotides at the −1, −2 and −3 positions of the 5′-handle, and a fixed number of PAM sequences.

Many 5′-handle mutants show a lack of resistance despite the presence of a *bona fide* PAM in the target and irrespective of the base pairing pattern (Figure S5). Efficient CRISPR-interference requires the presence of a cytosine at the −2 position of the crRNA repeat (Figure 4). Substitution of this position to guanidine or uracil interferes with CRISPR-defense. When this position is mutated to an adenosine, a partially resistant phenotype is observed during phage infection in conjunction with the canonical PAM, which is bound with the highest affinity by Cascade *in vitro*. Presumably this high affinity binding can compensate for the negative effects on DNA binding caused by mutations at the −2 position of the 5′-handle, leading to a partially phage resistant phenotype. Furthermore, CRISPR-mediated phage resistance requires a cytosine at the −3 position. The most likely explanation for the fact that some repeat mutants are not tolerated is that the Cascade subunits involved in binding the 5′-handle exhibit a level of sequence specificity.

Although combinations of fully complementary 5′-handles and protospacer flanking sequences do not lead to phage resistance *in vivo*, this appears to be base pairing independent (Figure S5), as restoring the wild-type base pairing pattern by altering protospacer flanking sequences fails to rescue the phage-sensitive phenotype. For example, the g8^C-3A, C-2T^ CRISPR fails to provide resistance either against M13 phage with a fully complementary CAT PAM (Figure 4B) or against a CTC PAM mutant phage, which is complementary at the −1 position only (Figure 4C). A similar result is obtained when g8^C-3A, C-2A^ CRISPR expressing cells are infected with CTT or CTC PAM phages (Figure 4D and E), indicating that the repeat sequence itself is affecting CRISPR-interference in these instances. Altogether, these data exclude the possibility that the Type I-E system makes use of a differential base pairing mechanism to inhibit self-targeting. The finding that the specificity of PAM recognition is unaffected by its potential to base pair with the 5′-handle is consistent with Cse1 being the only factor involved in PAM recognition [44].

To rule out the possibility that the specificity of PAM recognition by g8-Cascade variants depends on the expression levels of CRISPR-Cas components, the same analyses were performed with an engineered M13 targeting *E. coli* strain with *cas* genes fused to inducible promoters [12]. When repeat mutations were introduced into the genomic CRISPR cassette in this strain, identical results were obtained (Figure S6), showing that the data described here are expression level independent.

Previous studies on the *S. thermophilus* Type II-A CRISPR1/Cas system have revealed differences in PAM specificity and effectivity in either plasmid or phage interference assays [30],[45]. To test whether the Type I-E CRISPR/Cas system also displays assay-dependent differences in PAM utilization, we generated plasmids carrying the g8 protospacer (pG8) flanked by any of the 26 PAM mutants tested in the phage assays. Transformation of the pG8 variants into *E. coli* cells expressing Cascade, a g8 crRNA and Cas3 show that the four PAMs (CAT, CTT, CCT, and CTC) that provide interference during phage infection also affect plasmid transformation (resulting in a more than 1000-fold decrease in efficiency of transformation (e.o.t.)). Apart from these four PAMs, a non-consensus TTT PAM also yields a full resistance phenotype (Figure S7; >1000-fold decrease in e.o.t.), as has been observed before [8], while M13 phage carrying this non-consensus TTT PAM sequence escape interference (Figure 4A). In addition, ten non-consensus PAMs give rise to a partial resistance phenotype (Figure S7; e.o.t. <10^−1^ for CCA, CAA, GAT, CTG, and AGA PAMs; e.o.t. <10^−2^ for CTA, GTT, TAT, ATT and TTC PAMs), which is in line with previously reported partial resistance in *S. thermophilus* against transformation with a target plasmids carrying non-consensus PAMs [30]. The data show that PAM authentication during CRISPR-based protection is more promiscuous during plasmid transformation than during phage infection.

## Discussion

CRISPR-Cas systems are the only prokaryotic adaptive immune systems described to date. Although initially thought of as a single system, we now know that these systems are structurally and mechanistically diverse. Here we have investigated whether a differential base pairing mechanism to discriminate self from non-self, as described for the Type III-A system of *S. epidermidis*, also applies to the Type I-E CRISPR-Cas system of *E. coli* K12. By systematically mutating the crRNA repeat sequence and the PAM positions, we demonstrate that this Type I-E system does not utilize the potential for base pairing between the 5′-handle and the protospacer flanking sequences to avoid self targeting.

The −1 position of crRNA has recently been shown to be invader-derived and hence invariably has the potential to base pair with cognate DNA, both in *E. coli* [8], [11], [42] and in *S. thermophilus* [45], [46]. This discovery suggested that base pairing at the −1 position would be critical for target recognition by Cascade, in the same way that nucleotides in the seed region (nucleotides +1 to +5, +7 and +8) are essential for target recognition [41]. However, our results clearly show that base pairing at position −1 is not essential for CRISPR-interference. It has recently been suggested that the −1 position of the CRISPR repeat could be considered part of the spacer [42]. However, this does not seem appropriate since this nucleotide does not appear to be involved in base pairing with the invading target sequence. The absence of a base pairing requirement for the −1 position might suggest that this position is not available for base pairing due to structural constraints.

The −2 position of the crRNA repeat requires the presence of a cytosine for efficient CRISPR-interference (Figure 4). When this position is mutated to an adenosine, a partially resistant phenotype is observed during phage infection in conjunction with the canonical PAM. Substitution of the −2 position to a guanidine or uracil renders the CRISPR-interference pathway non-functional. Interestingly, mutation of the −2 position to adenosine causes an apparent structural alteration of the Cascade complex. While most subunits are present in the same apparent stoichiometry in the mutant g8^C-2A^-Cascade as in the wild-type complex, the Cse1 subunit is underrepresented. This might suggest that Cse1 interacts with the −2 position of the repeat and that interaction with this base is important for efficient incorporation of Cse1 into the complex. Like the −2 position, the −3 position requires a cytosine for CRISPR-mediated phage resistance to be manifested. However, complex formation is unaffected in g8^C-3G^-Cascade (Figure S4A).

The −3, −2 and −1 positions are among the most conserved bases of type 2 repeats [37]. Although the current resolution of the Cascade structure does not allow us to confidently pinpoint the location of the −2 and −3 bases of the 5′-handle of the crRNA, these bases appear to be part of a 5′ hook-like structure that is primarily cradled by the last subunit of the Cas7 hexamer (i.e., Cas7_6_) [47]. The arch of the crRNA may position the 5′ terminal nucleotides within bonding distance to residues in loop-1 of Cse1, which is consistent with the assembly defects reported for L1 mutations [44]. However, the resolution of the current Cascade structure and absence of density for L1 in the X-ray crystal structures of Cse1 prevent confident assignment of these interactions. Higher-resolution structures of the Cascade will be critical for a precise understanding how the crRNA and the Cas proteins are arranged in this complex.

In some CRISPR systems PAM sequences play an important role during different stages of CRISPR defense. In the Type I-E system of *E. coli*, PAM sequences are recognized by Cas1 and/or Cas2 during the selection of pre-spacers for integration into the CRISPR [9]. PAM motifs allow the CRISPR adaptation machinery to correctly orient newly acquired spacers into the CRISPR array [38], [48]–[50]. Interestingly, in Type I-E systems, the PAM selectivity of the CRISPR-adaptation machinery has co-evolved with that of the CRISPR-interference machinery, as the preference for the CTT PAM is observed both during Cas1/Cas2-dependent spacer integration [9] and during target DNA binding by Cascade [29]. In contrast, the *E. coli* I-F integration machinery appears to select for a PAM that overlaps but differs from the motif that yields optimal interference levels [43]. In this *E. coli* I-F subtype the PAM was found to be a GG motif at the −1 and −2 positions relative to the protospacer, while an overlapping, but different, motif (GG at the −2 and −3 positions) provided optimal interference levels [43]. The presence of a G at position −2 was both required and sufficient for interference. The I-F subtype of *Pectobacterium atrosepticum* on the other hand requires a GG motif immediately flanking the protospacer for interference, and mutagenesis of the G at position −1 to a T (which potentially base pairs with the repeat) gives rise to an escape phenotype [35]. Recently, a new nomenclature has been proposed that takes into account the differences in motif selectivity during spacer integration and CRISPR-interference [51].

PAMs have been shown to be important for CRISPR interference in various Type I and Type II CRISPR-Cas subtypes (e.g. Type I-A systems in *S. solfataricus* [40], Type I-B in *Haloferax volcanii* [39], Type I-E in *E. coli* [29], Type I-F in *P. aeruginosa* [52], *E. coli* [43] and *P. atrosepticum* [35], as well as in Type II-A and II-B systems of *Streptococcus pyogenes* and *S. thermophilus* [27], [30], [50], [53], [54]). Recently published x-ray crystal structures of the Cse1 subunit of Cascade [44], [55] have provided detailed insights into the molecular mechanism of Cascade-mediated recognition of the PAM. The well-conserved L1 loop of Cse1 was shown to directly interact with the PAM sequence and to enhance target DNA affinity in the presence of a *bona fide* PAM [44]. As such, the Cse1 subunit plays a crucial role in PAM authentication in Type I-E systems [44]. Our data indicate that PAM authentication occurs without the formation of base pairs between the 5′ handle of the crRNA and the PAM.

While Cascade-like complexes appear to be common components of Type I systems, the PAM-authenticating protein, Cse1, is unique to Type I-E systems. This could mean that other Cascade-like complexes, such as the aCascade (IA-Cascade) [25], IC-Cascade [17] the as yet unidentified ID-Cascade, and the Csy-complex (IF-Cascade) [26] may have their own specialized PAM-sensing proteins. It has been hypothesized that the large subunits of Type I systems (Cas8a1 and Cas8a2 (Type I-A), Cas8b (Type I-B), Cas8c (Type I-C), Cas10d (Type I-D), Cse1 (Type I-E), Csy1 (Type I-F)) are homologous to Cas10 proteins associated with the Type III systems [56], but these predictions await experimental verification. If these predictions are correct they may suggest that PAM recognition is carried out by the large subunit of other CRISPR-Cas subtypes.

**Figure 5:**
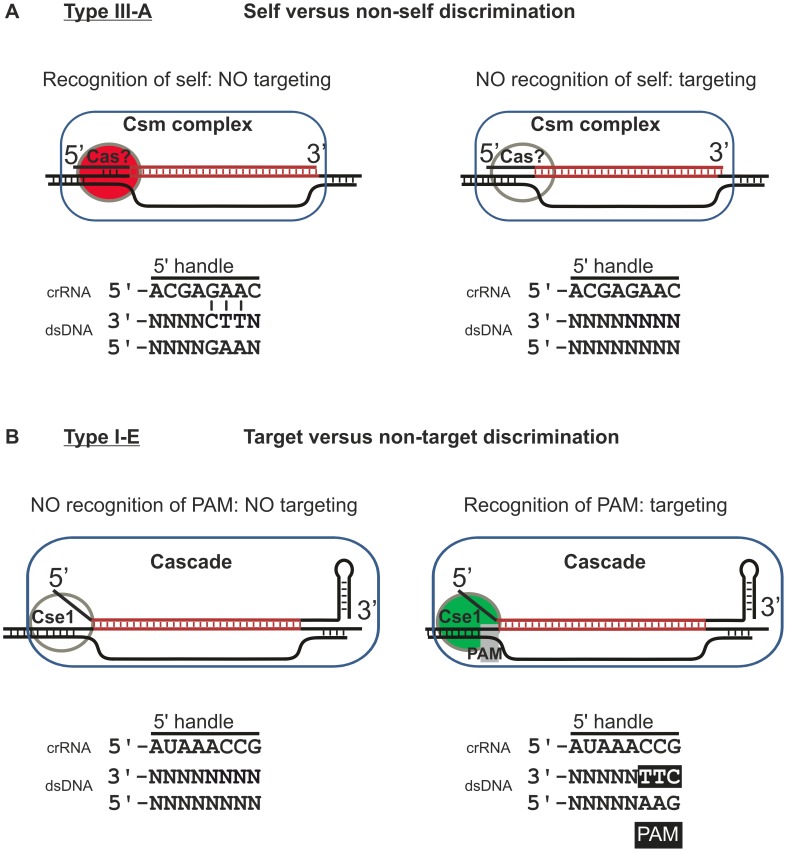
Model of self versus non-self discrimination by Type III-A systems and target versus non-target discrimination by Type I-E systems. **A**) Model of the mechanism employed by Type III-A systems. Type III-A systems presumably target DNA through R-loop formation. The crRNA (here depicted as the 37 nt species) could be part of a ribonucleoprotein complex consisting of Cas proteins and a single crRNA (Csm complex). R-loop formation and subsequent interference is inhibited when base pairing occurs between the −2, −3 and −4 positions of the repeat sequence at the 5′ end of the crRNA and the sequence flanking the 3′ end of the protospacer. How this base pairing is monitored is currently unknown, but it might involve a Cas protein. **B**) Model of target versus non-target discrimination by Type I-E systems. Type I-E systems target DNA through R-loop formation. The 61 nt crRNA is part of the Cascade ribonucleoprotein complex. R-loop formation and subsequent Cas3-mediated cleavage of the target DNA are activated when a PAM is present at the −1, −2 and −3 positions, in the sequence flanking the 3′ end of the protospacer. The presence of a PAM is monitored through the Cse1 subunit.

Under native-like expression levels, the change in affinity of Cascade for a target resulting from the presence or absence of a PAM sequence appears to be sufficient to serve as a robust mechanism to discriminate non-self target sequences (i.e. protospacers flanked by a PAM) from non-target sequences (i.e. protospacers without PAM) *in vivo* [44]. Given the absence of PAM sequences in the CRISPR array, self DNA automatically falls into the non-target category and is not subject to interference. For Type III systems, on the other hand, no PAMs have yet been found, suggesting that these systems lack PAMs [23], [36]. For Type III-A systems it has been shown that differentiation between self DNA and non-self DNA relies on sensing differential complementarity between the 5′-handle of the crRNA and the protospacer-flanking sequence (Figure 5A) [36]. This discrimination mechanism is based on specific recognition of self DNA, and is therefore best described by the term self versus non-self discrimination (Figure 5A). Here we demonstrate that self-avoidance by the Type I-E system does not rely on potential base pairing between crRNA repeats and protospacer flanking sequence. Therefore, Cascade lacks the ability to specifically recognize self and relies on specific target DNA recognition through PAM authentication. We argue that PAM authentication is a “target versus non-target” discrimination mechanism (Figure 5B), which is fundamentally different from the “self versus non-self” discrimination mechanism employed by Type III-A systems. Either mechanism is sufficient to avoid targeting of the CRISPR locus on the host genome. In target versus non-target discrimination, self sequences within the CRISPR locus (i.e. spacers) automatically belong to the non-target class, since PAM sequences are absent in the CRISPR repeat. Likewise, in self versus non-self discriminating systems target sequences fall in the non-self class. It appears likely that PAM-sensing CRISPR-Cas systems all make use of target versus non-target discrimination. Unlike Type III systems, discrimination between targets and non-targets by Type I-E systems cannot take place in the absence of a PAM.

Both discrimination mechanisms, however, are not mutually exclusive. The Type I-F system of *E. coli* LF82 has been speculated to utilize both target versus non-target discrimination and self versus non-self discrimination [43], although this hypothesis awaits experimental verification by testing the effect of crRNA repeat mutagenesis on CRISPR interference. By having both mechanisms in place an additional level of security against self-targeting of the host genome could be warranted. The requirement for a more stringent protection against self-targeting could be related to the constitutive gene expression of the Type I-F in *E. coli* LF82 [43], whereas the expression of the Type I-E system of *E. coli* K12 is repressed under laboratory growth conditions [57], [58], [59].

The distinct mechanisms of self versus non-self discrimination of Type III-A and target versus non-target recognition of Type I-E have implications for the route that invaders can take to escape CRISPR-interference. While both systems can be evaded by making point mutations in the protospacer [41], [60], only the Type I-E system can be evaded by mutations outside the protospacer, specifically in the region containing the PAM. In contrast, escape from Type III-A interference through mutations outside the protospacer seems rather unlikely, as it would typically require three mutations to establish base pairing between the 5′ handle and the protospacer flank [36].

## Materials and Methods

### Bacterial strains, gene cloning, plasmids and vectors

*E. coli* BL21 (DE3) strains were used for Cascade purification. Novablue (DE3) cells supplemented with CRISPR plasmid and plasmids expressing *cas* genes and engineered K12 strains with *cas* genes fused to inducible promoters were used for phage sensitivity tests and transformation assays. A description of the plasmids and the strains used in this study can be found in the Supplementary Information (Table S1).

### Protein expression and purification

Wildtype M13-Cascade was expressed in *E. coli* BL21 (DE3) and purified as described before [29], from pWUR408, pWUR514 and pWUR615 (Table S1). g8^G-1T^-Cascade, g8^C-2A^-Cascade, g8^C-3G^-Cascade, were expressed from pWUR408, pWUR514 and either pWUR680, pWUR682, or pWUR684, respectively (Table S1). pWUR680, pWUR682, and pWUR684 were generated by subcloning a synthetic CRISPR (Table S3 and Table S4, Geneart) into pACYC using EcoNI and Acc65I restriction sites. Although BL21 (DE3) contains genomic CRISPR loci, previous analyses by Mass Spectrometry have demonstrated that these expression and purification conditions yield homogeneous Cascade complexes loaded with crRNA species from the overexpression plasmids, and not from the chromosme [22].

### Gel electrophoresis

Purified Cascade was separated on a 12% SDS-PAGE as described before [22], and stained using Coomassie Blue overnight, followed by destaining in Millipore water. Nucleic acids were isolated from purified Cascade complexes using an extraction with phenol∶chloroform∶isoamylalcohol (25∶24∶1) equilibrated at pH 8.0 (Fluka) and separated on a 6M urea 15% acrylamide gel, as described in [22], followed by staining with SybR safe (Invitrogen) in a 1∶10000 dilution in TAE for 30 minutes. Electrophoretic Mobility Shift Assays were performed as in [29], using the PAGE-purified oligonucleotides listed in Table S2, which were annealed and 5′-labeled with ^32^P γ-ATP (PerkinElmer) using T4 polynucleotide kinase (Fermentas). Determining the Kd of the Cascade target DNA interaction was performed as described in [41]. Briefly, the signals of unbound and bound probe were quantified using Quantity One software (Bio-Rad). The fraction of bound probe was plotted against the total Cascade concentration, and the data fitted by nonlinear regression analysis to the following equation: Fraction bound probe = [Cascade]total/(Kd+[Cascade]total).

### Phage M13 mutagenesis

Mutations of PAM sequence preceding the g8 protospacer were introduced into the M13 phage genome by QuickChange Site-Directed Mutagenesis Kit (Stratagene) as described previously ([41]).

### CRISPR repeat mutagenesis

Repeat mutant library was generated by QuikChange Site-Directed Mutagenesis Kit (Stratagene) according to manufacturer's protocol. The g8 CRISPR cassette plasmid targeting the M13 phage gene 8 (pWUR477-g8, described in [41]) was used as template. Mutations were introduced at positions −3, −2, or −1 of the repeat preceding the g8 spacer.

### Phage infection studies

Cells sensitivity to wildtype and mutant M13 phages was determined by a spot test method as described [41] or using standard plaquing assay. Efficiency of plaquing was calculated as a ratio of the plaque number formed on a lawn of tested cells to the number of plaques on sensitive (non-targeting) cell lawn.

### Transformation assay

K12 strains with *cas* genes fused to inducible promoters and g8 spacer in CRISPR were transformed with 10 ng of plasmid DNA by electroporation. Transformation efficiency was determined as colony forming units for transformants of targeting strain BW40119 (Table S1) per µg DNA. Plasmids containing the g8 protospacer and PAM mutants were ordered synthetically at Geneart, Germany.

## Supporting Information

Figure S1 Shows the original EMSAs belonging to Figure 1. From left to right, lanes contain 600, 250, 120, 60, 25, 12.5, 6, 2.5, and 0 nM Cascade.
(TIF)

Figure S2 **A**) and **B**) show the original EMSAs belonging to Figure 2. From left to right, lanes contain 600, 250, 120, 60, 25, 12.5, 6, 2.5, and 0 nM Cascade.
(TIF)

Figure S3 **A**) and **B**) show the original EMSAs belonging to Figure 3. From left to right, lanes contain 600, 250, 120, 60, 25, 12.5, 6, 2.5, and 0 nM Cascade.
(TIF)

Figure S4 **A**) Coomassie stained SDS-PAGE of the WT and mutant g8-Cascade complexes shows that all complexes are formed with a correct stoichiometry. **B**) Nucleic acids bound to each of the g8-Cascade complexes shows that intact crRNA is present in all complexes.
(TIF)

Figure S5 Base pairing potential between the PAM mutants and the g8 CRISPR repeat mutants shown in Figure 4. A selection of the data shown in Figure 3 is shown, lacking the repeat/non-allowed PAM combinations. The repeat/allowed PAM combinations are highlighted that would give rise to a base pairing pattern corresponding to that observed for the resistant phenotype, but that do not give rise to CRISPR-interference. This suggests that in these cases the repeat sequence is interferes with CRISPR immunity rather than that a correlation exists with base pairing potential.
(TIF)

Figure S6 Cells sensitivity to wildtype and mutant M13 phages was determined using standard plaquing assay. Repeat mutations were introduced into the genomic CRISPR cassette in an engineered M13 targeting *E. coli* strain with *cas* genes fused to the inducible promoters [11]. Efficiency of plaquing was calculated as a ratio of the plaque number formed on a lawn of tested cells to the number of plaques on sensitive (nontargeting) cell lawn.
(TIF)

Figure S7 Efficiency of transformation of *E. coli* expressing Cascade, g8-crRNA and Cas3 with pG8 plasmid variants carrying the g8 protospacer flanked by 26 different PAM variants. Efficiency of transformation was calculated as the number of transformants per microgram DNA. Plasmid pUC19 serves as a negative control. The CGG PAM (indicated with #) corresponds to the repeat sequence flanking a spacer in the CRISPR array. The four PAM sequences that provide a phage resistant phenotype are indicated with an asterisk (*). The TTT PAM provides resistance against plasmid transformation, but not against phage infection.
(TIF)

Table S1 Putative basepairing between PAM and crRNA 5′ or 3′ handles in various subtypes.
(DOC)

Table S2 Plasmids and strains used in this study.
(DOC)

Table S3 Oligo's used in this study.
(DOC)

Table S4 Synthetic CRISPR sequences used in this study.
(DOC)
