# Supplementary figures and images for: Type I-E CRISPR-Cas Systems Discriminate Target from Non-Target DNA through Base Pairing-Independent PAM Recognition
Source: PLoS Genet. 2013 Sep 5;9(9):e1003742. doi: 10.1371/journal.pgen.1003742 (PMC3764190; doi:10.1371/journal.pgen.1003742)

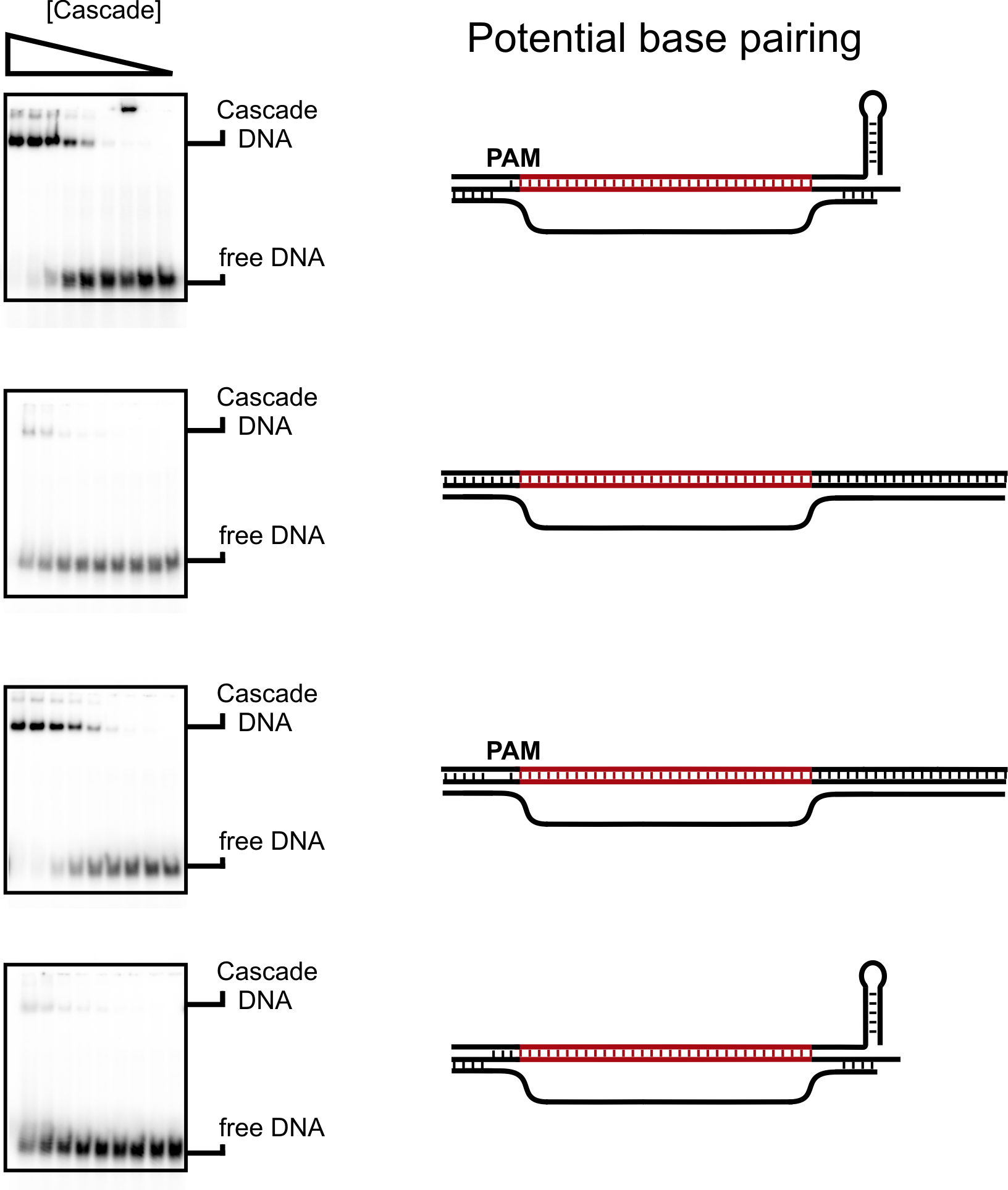

Supplement: Figure S1 — Shows the original EMSAs belonging to Figure 1. From left to right, lanes contain 600, 250, 120, 60, 25, 12.5, 6, 2.5, and 0 nM Cascade. (TIF) [file pgen.1003742.s001.tif]

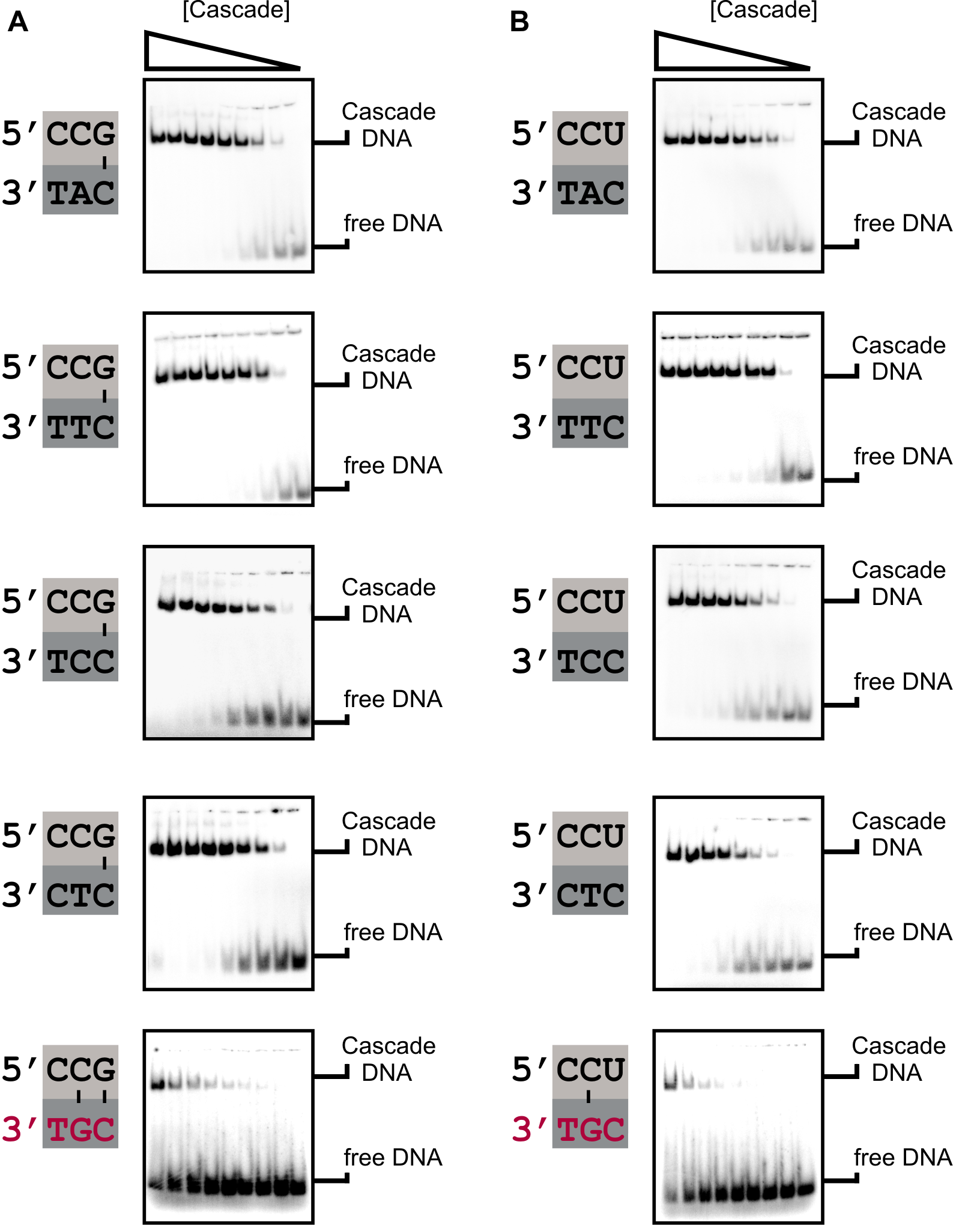

Supplement: Figure S2 — A) and B) show the original EMSAs belonging to Figure 2. From left to right, lanes contain 600, 250, 120, 60, 25, 12.5, 6, 2.5, and 0 nM Cascade. (TIF) [file pgen.1003742.s002.tif]

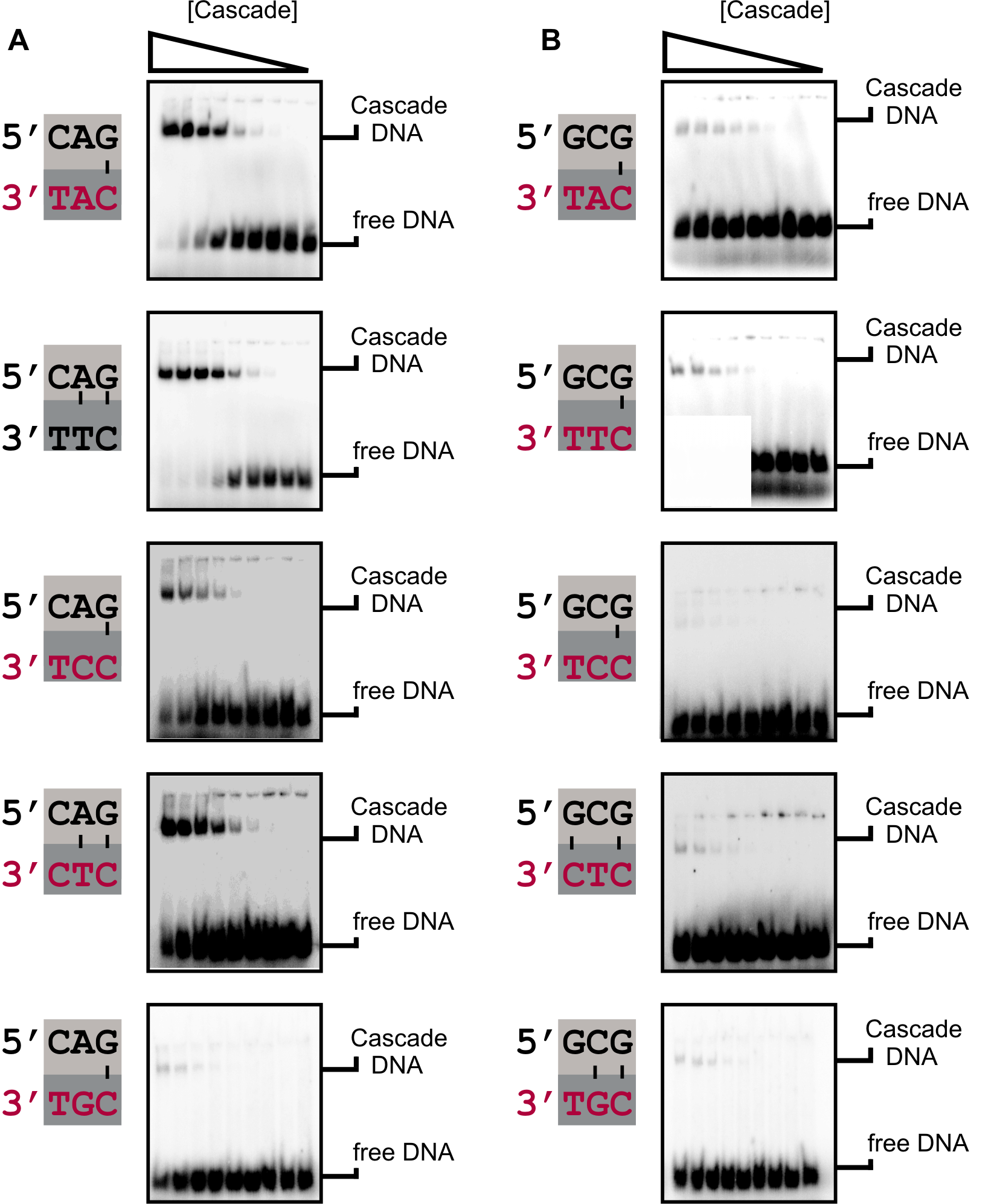

Supplement: Figure S3 — A) and B) show the original EMSAs belonging to Figure 3. From left to right, lanes contain 600, 250, 120, 60, 25, 12.5, 6, 2.5, and 0 nM Cascade. (TIF) [file pgen.1003742.s003.tif]

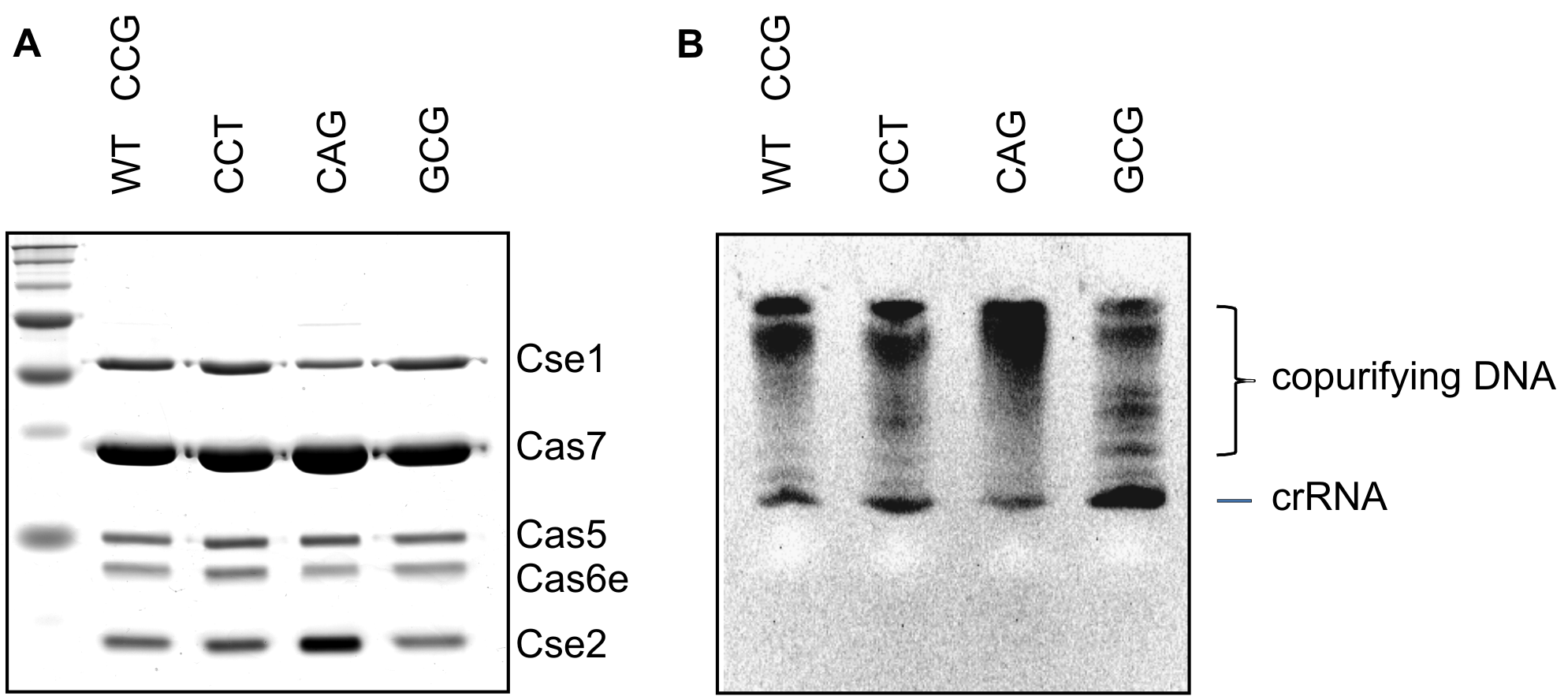

Supplement: Figure S4 — A) Coomassie stained SDS-PAGE of the WT and mutant g8-Cascade complexes shows that all complexes are formed with a correct stoichiometry. B) Nucleic acids bound to each of the g8-Cascade complexes shows that intact crRNA is present in all complexes. (TIF) [file pgen.1003742.s004.tif]

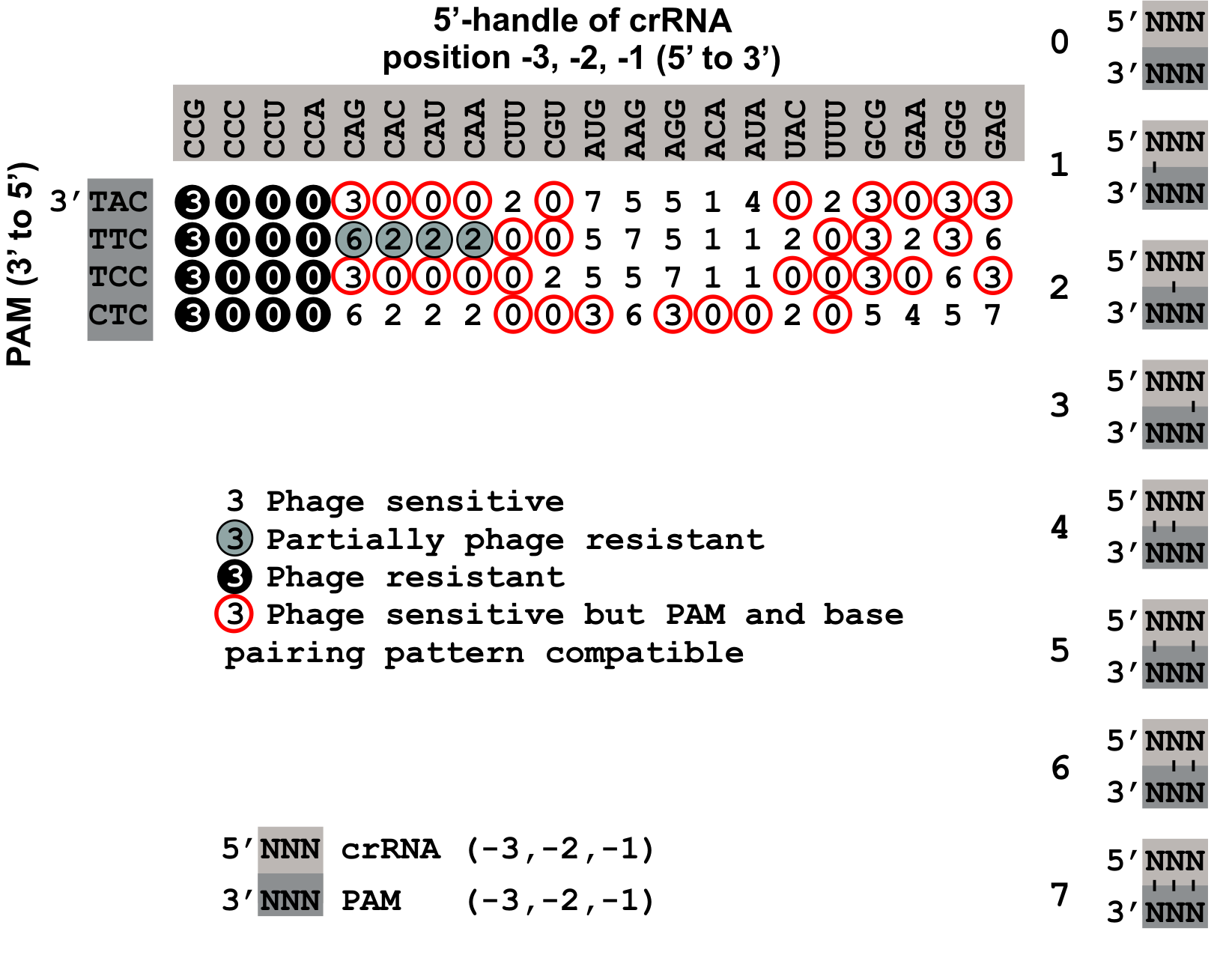

Supplement: Figure S5 — Base pairing potential between the PAM mutants and the g8 CRISPR repeat mutants shown in Figure 4. A selection of the data shown in Figure 3 is shown, lacking the repeat/non-allowed PAM combinations. The repeat/allowed PAM combinations are highlighted that would give rise to a base pairing pattern corresponding to that observed for the resistant phenotype, but that do not give rise to CRISPR-interference. This suggests that in these cases the repeat sequence is interferes with CRISPR immunity rather than that a correlation exists with base pairing potential. (TIF) [file pgen.1003742.s005.tif]

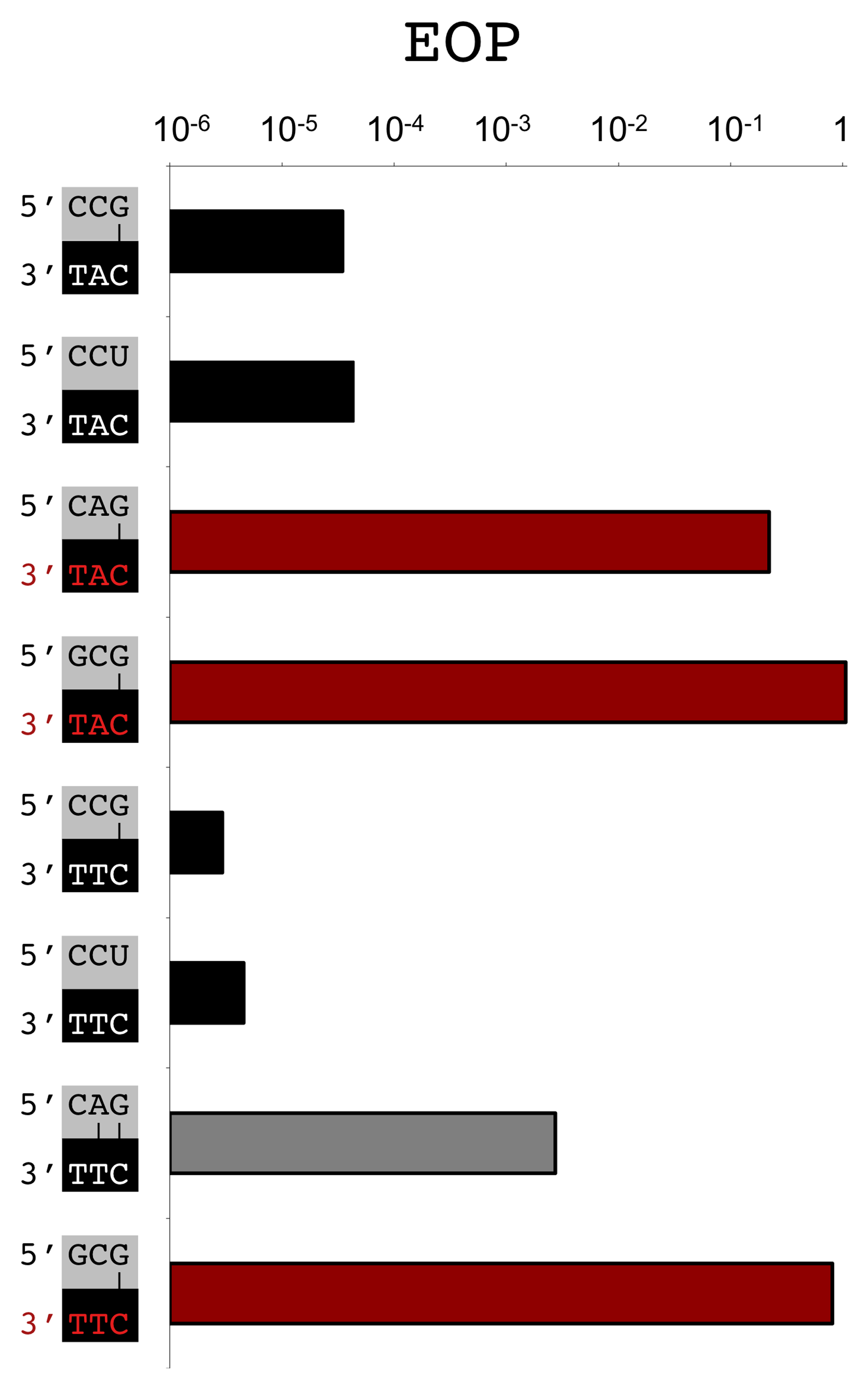

Supplement: Figure S6 — Cells sensitivity to wildtype and mutant M13 phages was determined using standard plaquing assay. Repeat mutations were introduced into the genomic CRISPR cassette in an engineered M13 targeting E. coli strain with cas genes fused to the inducible promoters [11]. Efficiency of plaquing was calculated as a ratio of the plaque number formed on a lawn of tested cells to the number of plaques on sensitive (nontargeting) cell lawn. (TIF) [file pgen.1003742.s006.tif]

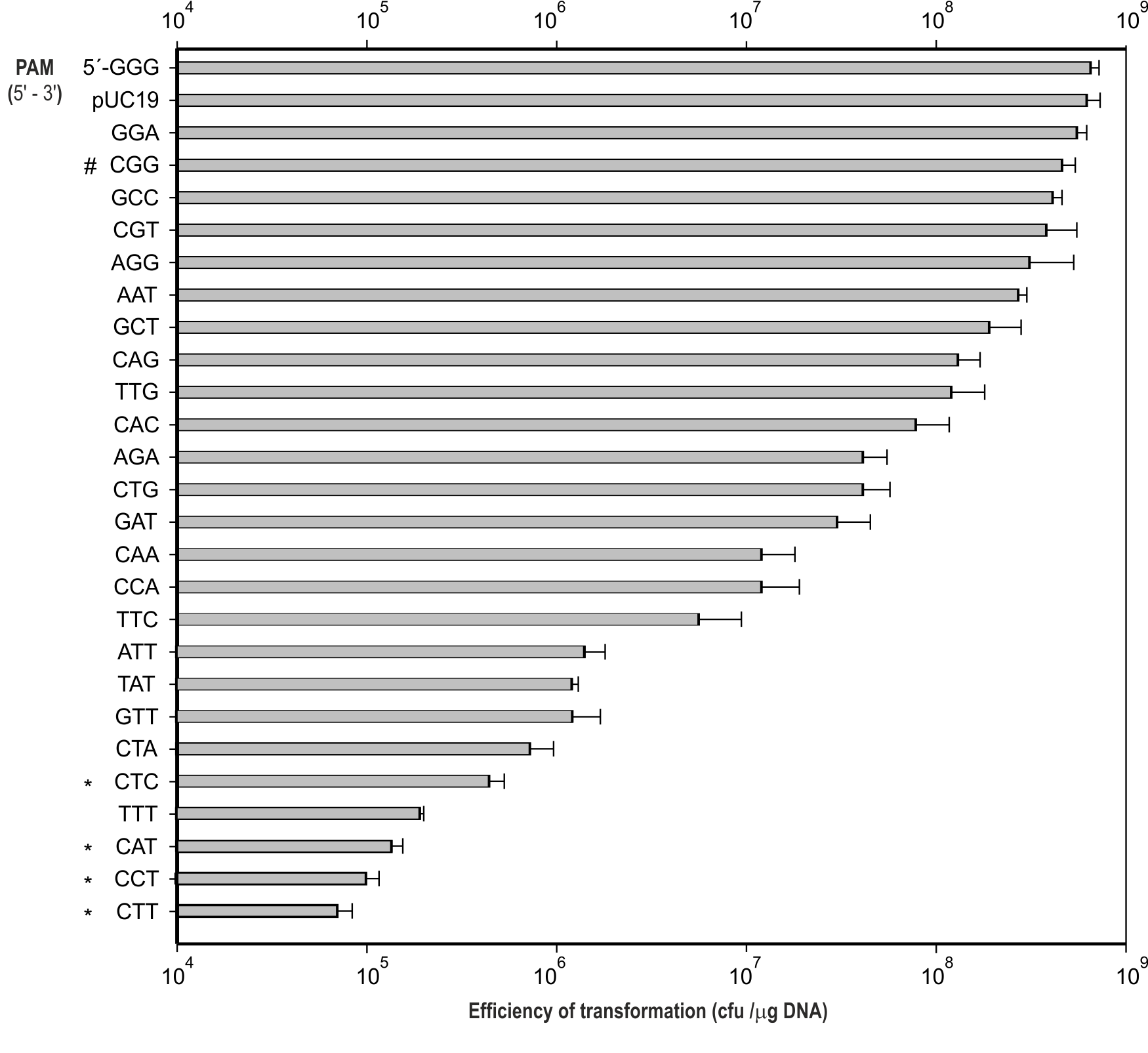

Supplement: Figure S7 — Efficiency of transformation of E. coli expressing Cascade, g8-crRNA and Cas3 with pG8 plasmid variants carrying the g8 protospacer flanked by 26 different PAM variants. Efficiency of transformation was calculated as the number of transformants per microgram DNA. Plasmid pUC19 serves as a negative control. The CGG PAM (indicated with #) corresponds to the repeat sequence flanking a spacer in the CRISPR array. The four PAM sequences that provide a phage resistant phenotype are indicated with an asterisk (*). The TTT PAM provides resistance against plasmid transformation, but not against phage infection. (TIF) [file pgen.1003742.s007.tif]
