# Supplementary material for: Type I-E CRISPR-Cas Systems Discriminate Target from Non-Target DNA through Base Pairing-Independent PAM Recognition
Source: PLoS Genet. 2013 Sep 5;9(9):e1003742. doi: 10.1371/journal.pgen.1003742 (PMC3764190; doi:10.1371/journal.pgen.1003742)
Supplement: Table S2 — Plasmids and strains used in this study. (DOC) [file pgen.1003742.s009.doc]

| **Plasmids** | **Description and order of genes (5’-3’)** | **Restriction sites** | **Source** |
| --- | --- | --- | --- |
| pWUR397 | *cas3* in pRSF-1b, no tags |  |  |
| pWUR399 | *cse1-cse2-cas7-cas5-cas6e-cas1-cas2* in pCDF-1b, no tags |  |  |
| pWUR400 | *cse1-cse2-cas7-cas5-cas6e* in pCDF-1b, no tags |  |  |
| pWUR408 | *cse1* in pRSF-1b, no tags |  |  |
| pWUR477 | non targeting CRISPR in pACYCDuet-1 |  |  |
| pWUR477-g8 | M13-CRISPR containing spacer g8 cloned into the EcoRI and BamHI sites of pWUR477 |  |  |
| pWUR514 | *cse2* with Strep-tag II (N-term)-*cas7*-*cas5-cas6e* in pET52b |  |  |
| pWUR615 | M13-CRISPR containing spacer g8 | EcoNI/Acc65I |  |
| pWUR680 | M13^G-1T^ -CRISPR containing spacer g8, corresponding to the EcoNI/Acc65I digest of insert M13G-1T (Table S3) in pACYCDuet-1 | EcoNI/Acc65I | This study |
| pWUR682 | M13^C-2A^-CRISPR containing spacer g8, corresponding to the EcoNI/Acc65I digest of insert M13C-2A (Table S3) cloned into pACYCDuet-1 | EcoNI/Acc65I | This study |
| pWUR684 | M13^C-3G^-CRISPR containing spacer g8, corresponding to the EcoNI/Acc65I digest of insert M13C-3G (Table S3) in pACYCDuet-1 | EcoNI/Acc65I | This study |
| pWUR687 | BG4064/BG4065 in pUC19 | SmaI | This study |
| pWUR688 | BG4066/BG4067 in pUC19 | SmaI | This study |
| pWUR689 | BG4068/BG4069 in pUC19 | SmaI | This study |
| pWUR690 | BG4070/BG4071 in pUC19 | SmaI | This study |

| **Strains** | **Description** | **Source** |
| --- | --- | --- |
| BW40119 | K12 F’, *lac*UV5-*cas3* *cat*:: *araBp*8-*casA,* g8 spacer in CRISPR I |  |
| KD163 | K12 F’, *lac*UV5-*cas3* *araBp*8-*casA,* Repeat G-1T, g8 spacer in CRISPR I | This study |
| KD160 | K12 F’, *lac*UV5-*cas3* *araBp*8-*casA,* Repeat C-2A, g8 spacer in CRISPR I | This study |
| KD157 | K12 F’, *lac*UV5-*cas3* *araBp*8-*casA,* Repeat C-3G, g8 spacer in CRISPR I | This study |

**References**

1. Datsenko KA, Pougach K, Tikhonov A, Wanner BL, Severinov K, et al. (2012) Molecular memory of prior infections activates the CRISPR/Cas adaptive bacterial immunity system. Nat Commun 3: 945.

2. Mojica FJM, Diez-Villasenor C, Garcia-Martinez J, Almendros C (2009) Short motif sequences determine the targets of the prokaryotic CRISPR defence system. Microbiology-Sgm 155: 733-740.

3. Lillestol RK, Shah SA, Brugger K, Redder P, Phan H, et al. (2009) CRISPR families of the crenarchaeal genus Sulfolobus: bidirectional transcription and dynamic properties. Mol Microbiol 72: 259-272.

4. Gudbergsdottir S, Deng L, Chen Z, Jensen JV, Jensen LR, et al. (2011) Dynamic properties of the Sulfolobus CRISPR/Cas and CRISPR/Cmr systems when challenged with vector-borne viral and plasmid genes and protospacers. Mol Microbiol 79: 35-49.

5. Fischer S, Maier LK, Stoll B, Brendel J, Fischer E, et al. (2012) An archaeal immune system can detect multiple protospacer adjacent motifs (PAMs) to target invader DNA. J Biol Chem.

6. Semenova E, Nagornykh M, Pyatnitskiy M, Artamonova, II, Severinov K (2009) Analysis of CRISPR system function in plant pathogen Xanthomonas oryzae. FEMS Microbiol Lett 296: 110-116.

7. Semenova E, Jore MM, Datsenko KA, Semenova A, Westra ER, et al. (2011) Interference by clustered regularly interspaced short palindromic repeat (CRISPR) RNA is governed by a seed sequence. Proc Natl Acad Sci U S A 108: 10098-10103.

8. Swarts DC, Mosterd C, van Passel MW, Brouns SJ (2012) CRISPR Interference Directs Strand Specific Spacer Acquisition. PloS one 7: e35888.

9. Westra ER, van Erp PB, Kunne T, Wong SP, Staals RH, et al. (2012) CRISPR Immunity Relies on the Consecutive Binding and Degradation of Negatively Supercoiled Invader DNA by Cascade and Cas3. Mol Cell 46: 595-605.

10. Horvath P, Barrangou R CRISPR/Cas, the immune system of bacteria and archaea. Science 327: 167-170.

11. Sinkunas T, Gasiunas G, Waghmare SP, Dickman MJ, Barrangou R, et al. (2013) In vitro reconstitution of Cascade-mediated CRISPR immunity in Streptococcus thermophilus. EMBO J.

12. Cady KC, Bondy-Denomy J, Heussler GE, Davidson AR, O'Toole GA (2012) The CRISPR/Cas adaptive immune system of Pseudomonas aeruginosa mediates resistance to naturally occurring and engineered phages. J Bacteriol 194: 5728-5738.

13. Cady KC, White AS, Hammond JH, Abendroth MD, Karthikeyan RS, et al. (2011) Prevalence, conservation and functional analysis of Yersinia and Escherichia CRISPR regions in clinical Pseudomonas aeruginosa isolates. Microbiology 157: 430-437.

14. Almendros C, Guzman NM, Diez-Villasenor C, Garcia-Martinez J, Mojica FJ (2012) Target Motifs Affecting Natural Immunity by a Constitutive CRISPR-Cas System in Escherichia coli. PLoS One 7: e50797.

15. Horvath P, Romero DA, Coute-Monvoisin AC, Richards M, Deveau H, et al. (2008) Diversity, activity, and evolution of CRISPR loci in Streptococcus thermophilus. J Bacteriol 190: 1401-1412.

16. Lopez-Sanchez MJ, Sauvage E, Da Cunha V, Clermont D, Ratsima Hariniaina E, et al. (2012) The highly dynamic CRISPR1 system of Streptococcus agalactiae controls the diversity of its mobilome. Mol Microbiol.

17. Jinek M, Chylinski K, Fonfara I, Hauer M, Doudna JA, et al. (2012) A Programmable Dual-RNA-Guided DNA Endonuclease in Adaptive Bacterial Immunity. Science.

18. Marraffini LA, Sontheimer EJ (2010) Self versus non-self discrimination during CRISPR RNA-directed immunity. Nature 463: 568-571.

19. Hale CR, Majumdar S, Elmore J, Pfister N, Compton M, et al. (2012) Essential Features and Rational Design of CRISPR RNAs that Function with the Cas RAMP Module Complex to Cleave RNAs. Mol Cell 45: 292-302.

20. Zhang J, Rouillon C, Kerou M, Reeks J, Brugger K, et al. (2012) Structure and Mechanism of the CMR Complex for CRISPR-Mediated Antiviral Immunity. Mol Cell 45: 303-313.

21. Brouns SJJ, Jore MM, Lundgren M, Westra ER, Slijkhuis RJH, et al. (2008) Small CRISPR RNAs guide antiviral defense in prokaryotes. Science 321: 960-964.

22. Jore MM, Lundgren M, van Duijn E, Bultema JB, Westra ER, et al. (2011) Structural basis for CRISPR RNA-guided DNA recognition by Cascade. Nat Struct Mol Biol 18: 529-536.
