# Supplementary material for: Type I-E CRISPR-Cas Systems Discriminate Target from Non-Target DNA through Base Pairing-Independent PAM Recognition
Source: PLoS Genet. 2013 Sep 5;9(9):e1003742. doi: 10.1371/journal.pgen.1003742 (PMC3764190; doi:10.1371/journal.pgen.1003742)
Supplement: Table S3 — Oligo's used in this study. (DOC) [file pgen.1003742.s010.doc]

| name | sequence | characteristics |
| --- | --- | --- |
| BG3575  **gelpure** | GCTACCCTCGTTCCG**ATG**CTGTCTTTCGCTGCTGAGGGTGACGATCCCGCAAAAGCGGCCTTTAA | ATG PAM  M13 probe |
| BG3576 **gelpure** | TTAAAGGCCGCTTTTGCGGGATCGTCACCCTCAGCAGCGAAAGACAG**CAT**CGGAACGAGGGTAGC | ATG PAM  M13 probe |
| BG3577 **gelpure** | GCTACCCTCGTTCCG**AAG**CTGTCTTTCGCTGCTGAGGGTGACGATCCCGCAAAAGCGGCCTTTAA | AAG PAM  M13 probe |
| BG3578 **gelpure** | TTAAAGGCCGCTTTTGCGGGATCGTCACCCTCAGCAGCGAAAGACAG**CTT**CGGAACGAGGGTAGC | AAG PAM  M13 probe |
| BG3579 **gelpure** | GCTACCCTCGTTCCG**AGG**CTGTCTTTCGCTGCTGAGGGTGACGATCCCGCAAAAGCGGCCTTTAA | AGG PAM  M13 probe |
| BG3580 **gelpure** | TTAAAGGCCGCTTTTGCGGGATCGTCACCCTCAGCAGCGAAAGACAG**CCT**CGGAACGAGGGTAGC | AGG PAM  M13 probe |
| BG3581 **gelpure** | GCTACCCTCGTTCCG**GAG**CTGTCTTTCGCTGCTGAGGGTGACGATCCCGCAAAAGCGGCCTTTAA | GAG PAM  M13 probe |
| BG3582 **gelpure** | TTAAAGGCCGCTTTTGCGGGATCGTCACCCTCAGCAGCGAAAGACAG**CTC**CGGAACGAGGGTAGC | GAG PAM  M13 probe |
| BG3583 **gelpure** | GCTACCCTCGTTCCG**ACG**CTGTCTTTCGCTGCTGAGGGTGACGATCCCGCAAAAGCGGCCTTTAA | ACG PAM  M13 probe |
| BG3584 **gelpure** | TTAAAGGCCGCTTTTGCGGGATCGTCACCCTCAGCAGCGAAAGACAG**CGT**CGGAACGAGGGTAGC | ACG PAM  M13 probe |
| BG3585 **gelpure** | GCTACCCTCGTTCCG**CCC**CTGTCTTTCGCTGCTGAGGGTGACGATCCCGCAAAAGCGGCCTTTAA | CCC PAM  M13 probe |
| BG3586 **gelpure** | TTAAAGGCCGCTTTTGCGGGATCGTCACCCTCAGCAGCGAAAGACAG**GGG**CGGAACGAGGGTAGC | CCC PAM  M13 probe |
| BG3591 gelpure | GCTACCCTCGTTCCG**AAT**CTGTCTTTCGCTGCTGAGGGTGACGATCCCGCAAAAGCGGCCTTTAA | AAT PAM  **M13 probe** |
| BG3592 gelpure | TTAAAGGCCGCTTTTGCGGGATCGTCACCCTCAGCAGCGAAAGACAG**ATT**CGGAACGAGGGTAGC | AAT PAM  **M13 probe** |
| BG4064  Gelpure | GCTACCCTCGATAAACCGCTGTCTTTCGCTGCTGAGGGTGACGATCCCGCGAGTTCCCCGCGCCAGCGGGG | DISPLACED STRAND  full complementarity |
| BG4065  gelpure | CCCCGCTGGCGCGGGGAACTCGCGGGATCGTCACCCTCAGCAGCGAAAGACAGCGGTTTATCGAGGGTAGC | BASE PAIRING STRAND full complementarity |
| BG4066  Gelpure | GCTACCCTCGATAAA**ATG**CTGTCTTTCGCTGCTGAGGGTGACGATCCCGCGAGTTCCCCGCGCCAGCGGGG | DISPLACED STRAND full complementarity + PAM |
| BG4067  Gelpure | CCCCGCTGGCGCGGGGAACTCGCGGGATCGTCACCCTCAGCAGCGAAAGACAG**CAT**TTTATCGAGGGTAGC | BASE PAIRING STRAND full complementarity + PAM |
| BG4068  gelpure | GCTACCCTCGTATTTCCGCTGTCTTTCGCTGCTGAGGGTGACGATCCCGCCTCAAGGGGCGCGGTCGCCCC | DISPLACED STRAND no complementarity except PAM |
| BG4069  Gelpure | GGGGCGACCGCGCCCCTTGAGGCGGGATCGTCACCCTCAGCAGCGAAAGACAGCGGAAATACGAGGGTAGC | BASE PAIRING STRAND no complementarity except PAM |
| BG4070  Gelpure | GCTACCCTCGTATTT**ATG**CTGTCTTTCGCTGCTGAGGGTGACGATCCCGCCTCAAGGGGCGCGGTCGCCCC | DISPLACED STRAND no complementarity + PAM |
| BG4071  Gelpure | GGGGCGACCGCGCCCCTTGAGGCGGGATCGTCACCCTCAGCAGCGAAAGACAG**CAT**AAATACGAGGGTAGC | BASE PAIRING STRAND no complementarity + PAM |
