# Supplementary material for: Type I-E CRISPR-Cas Systems Discriminate Target from Non-Target DNA through Base Pairing-Independent PAM Recognition
Source: PLoS Genet. 2013 Sep 5;9(9):e1003742. doi: 10.1371/journal.pgen.1003742 (PMC3764190; doi:10.1371/journal.pgen.1003742)
Supplement: Table S4 — Synthetic CRISPR sequences used in this study. (DOC) [file pgen.1003742.s011.doc]

**G8G-1T** CACTATAGGGCGAATTGAAGGAAGGCCGTCAAGGCCGCATCCTGCATTAGGTAATACGACTCACTATAGGATAAACCTC

TGTCTTTCGCTGCTGAGGGTGACGATCCCGCGAGTTCCCCGCGCCAGCGGGGATAAACCTCTGTCTTTCGCTGCTGAGG

GTGACGATCCCGCGAGTTCCCCGCGCCAGCGGGGATAAACCTCTGTCTTTCGCTGCTGAGGGTGACGATCCCGCGAGTT

CCCCGCGCCAGCGGGGATAAACCTCTGTCTTTCGCTGCTGAGGGTGACGATCCCGCGAGTTCCCCGCGCCAGCGGGGAT

AAACCTCTGTCTTTCGCTGCTGAGGGTGACGATCCCGCGAGTTCCCCGCGCCAGCGGGGATAAACCTCTGTCTTTCGCT

GCTGAGGGTGACGATCCCGCGAGTTCCCCGCGCCAGCGGGGATAAACCTCTGTCTTTCGCTGCTGAGGGTGACGATCCC

GCGAGTTCCCCGCGCCAGCGGGGATAAACCTGGTACCCTGGGCCTCATGGGCCTTCCTTTCACTGCCCGCTTTCCAG

**G8C-2A** CACTATAGGGCGAATTGAAGGAAGGCCGTCAAGGCCGCATCCTGCATTAGGTAATACGACTCACTATAGGATAAACAGC

TGTCTTTCGCTGCTGAGGGTGACGATCCCGCGAGTTCCCCGCGCCAGCGGGGATAAACAGCTGTCTTTCGCTGCTGAGG

GTGACGATCCCGCGAGTTCCCCGCGCCAGCGGGGATAAACAGCTGTCTTTCGCTGCTGAGGGTGACGATCCCGCGAGTT

CCCCGCGCCAGCGGGGATAAACAGCTGTCTTTCGCTGCTGAGGGTGACGATCCCGCGAGTTCCCCGCGCCAGCGGGGAT

AAACAGCTGTCTTTCGCTGCTGAGGGTGACGATCCCGCGAGTTCCCCGCGCCAGCGGGGATAAACAGCTGTCTTTCGCT

GCTGAGGGTGACGATCCCGCGAGTTCCCCGCGCCAGCGGGGATAAACAGCTGTCTTTCGCTGCTGAGGGTGACGATCCC

GCGAGTTCCCCGCGCCAGCGGGGATAAACAGGGTACCCTGGGCCTCATGGGCCTTCCTTTCACTGCCCGCTTTCCAG

**G8C-3G** CACTATAGGGCGAATTGAAGGAAGGCCGTCAAGGCCGCATCCTGCATTAGGTAATACGACTCACTATAGGATAAAGCGC

TGTCTTTCGCTGCTGAGGGTGACGATCCCGCGAGTTCCCCGCGCCAGCGGGGATAAAGCGCTGTCTTTCGCTGCTGAGG

GTGACGATCCCGCGAGTTCCCCGCGCCAGCGGGGATAAAGCGCTGTCTTTCGCTGCTGAGGGTGACGATCCCGCGAGTT

CCCCGCGCCAGCGGGGATAAAGCGCTGTCTTTCGCTGCTGAGGGTGACGATCCCGCGAGTTCCCCGCGCCAGCGGGGAT

AAAGCGCTGTCTTTCGCTGCTGAGGGTGACGATCCCGCGAGTTCCCCGCGCCAGCGGGGATAAAGCGCTGTCTTTCGCT

GCTGAGGGTGACGATCCCGCGAGTTCCCCGCGCCAGCGGGGATAAAGCGCTGTCTTTCGCTGCTGAGGGTGACGATCCC

GCGAGTTCCCCGCGCCAGCGGGGATAAAGCGGGTACCCTGGGCCTCATGGGCCTTCCTTTCACTGCCCGCTTTCCAG
